# Enzyme-Instructed Aggregation/Dispersion of Fluorophores for Near-Infrared Fluorescence Imaging In Vivo

**DOI:** 10.3390/molecules28145360

**Published:** 2023-07-12

**Authors:** Zhipeng Zhang, Peiyao Chen, Yao Sun

**Affiliations:** 1 Xianning Medical College, Hubei University of Science & Technology, Xianning 437000, China; zzplbeckham@hbust.edu.cn; 2 Key Laboratory of Fermentation Engineering (Ministry of Education), National “111” Center for Cellular Regulation and Molecular Pharmaceutics, Hubei Key Laboratory of Industrial Microbiology, School of Food and Biological Engineering, Hubei University of Technology, Wuhan 430068, China; 3 National Key Laboratory of Green Pesticide, College of Chemistry, Central China Normal University, Wuhan 430079, China

**Keywords:** near-infrared fluorescence, enzyme, activatable probe

## Abstract

Near-infrared (NIR) fluorescence is a noninvasive, highly sensitive, and high-resolution modality with great potential for in vivo imaging. Compared with “Always-On” probes, activatable NIR fluorescent probes with “Turn-Off/On” or “Ratiometric” fluorescent signals at target sites exhibit better signal-to-noise ratio (SNR), wherein enzymes are one of the ideal triggers for probe activation, which play vital roles in a variety of biological processes. In this review, we provide an overview of enzyme-activatable NIR fluorescent probes and concentrate on the design strategies and sensing mechanisms. We focus on the aggregation/dispersion state of fluorophores after the interaction of probes and enzymes and finally discuss the current challenges and provide some perspective ideas for the construction of enzyme-activatable NIR fluorescent probes.

## 1. Introduction

Fluorescence imaging, a mighty molecular imaging modality with high sensitivity and spatial resolution, has been wildly used in the real-time visualization of biological processes [1]. However, conventional fluorescence probes emitting fluorescence in the visible region exhibit poor depth penetration, which fails the in vivo imaging [2]. Over the past decade, near-infrared (NIR) fluorescence probes with longer emission wavelengths show great potential for in vivo imaging of diverse biologically important species [3,4,5]. Typically, NIR fluorescence probes are advantageous in reducing light scattering, absorption, and auto-fluorescence interference from tissues, affording deeper penetration depth and improved signal-to-noise ratio (SNR) [6,7,8]. Due to low target-to-background ratios, probes with nonactivatable “Always-On” fluorescence signals are limited in their ability to detect small targeting regions. Generally, small molecular probes have low target accumulation, while nanostructure probes are cleared slowly and, thus, hold high background signals [9,10]. “Turn-Off/On” or “Ratiometric” fluorescent probes, on the other hand, are activated only at target sites to reduce the background signal, assuring the high quality of the imaging [11,12,13].

**Scheme 1:**
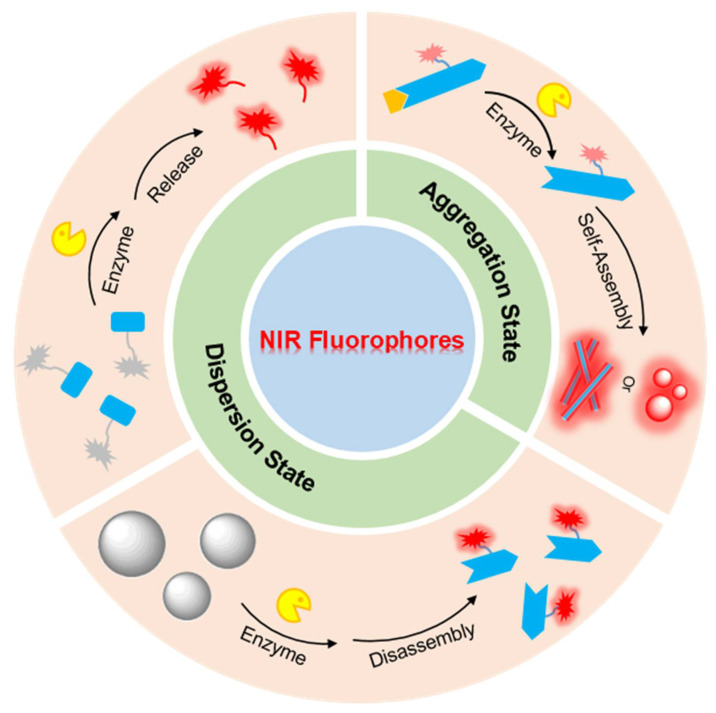
Schematic diagram of the strategies of enzyme-instructed aggregation/dispersion of NIR fluorophores.

Multiple stimuli could activate the fluorescence of probes, such as pH [14,15,16,17], reactive oxygen species (ROS) [18,19,20,21], glutathione (GSH) [22,23,24], or enzymes [25,26,27,28,29,30]. Among them, enzymes are one of the ideal targets to construct activatable fluorescent probes. Enzymes are known to play vital roles in numerous biological processes. Abnormal expressions of enzymes are closely associated with a variety of diseases [31,32,33]. For example, as a typical enzyme, β-galactosidase shows enhanced expression in senescence cells and primary ovarian cancer cells [34,35,36]. In addition, intracellular cysteine protease caspase-3/7 is closely associated with apoptosis, which could be activated via the extrinsic pathway and the intrinsic pathway, leading to rapid cell death [37]. The level of activated caspase-3/7 is positively correlated with the tumor therapeutic efficiency. Therefore, monitoring the activity of caspase-3/7 could be used for evaluating tumor response to therapy, such as chemotherapy [38], photothermal therapy (PTT) [39], radiotherapy [40], etc. Together with the aforementioned enzymes, a variety of enzymes have been exploited as potential triggers for activatable fluorescent probes, such as granzyme B [41,42,43,44], matrix metalloproteinases (MMPs) [45,46,47], and aminopeptidase N [48,49]. In this review, we summarize the common strategies for enzyme-activatable NIR fluorescence imaging based on the aggregation/dispersion state of fluorophores (Scheme 1). Subsequently, the mechanisms of the activation are also discussed. Finally, we provide some challenges and perspective ideas for the construction of new enzyme-activatable NIR fluorescent probes.

## 2. Enzyme-Instructed Release of Free NIR Fluorophores In Vivo

**Figure 1:**
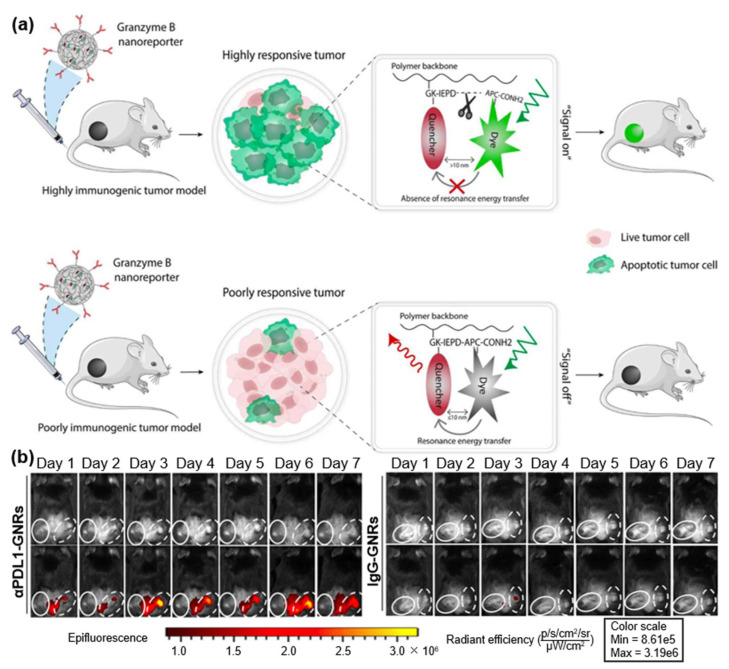
(**a**) Illustration of the mechanism of real-time monitoring of immunotherapy response using granzyme B nanoreporter **GNR**. (**b**) Representative bright-field (top row) and fluorescence (bottom row) images of the mice i.v. injected with **αPDL1-GNRs** or **IgG-GNRs**. Highly immunogenic MC38 tumors were indicated with dotted oval (right flanks), and poorly immunogenic B16/F10 tumors were indicated with solid oval (left flanks). Reproduced with permission [43]. Copyright 2020, American Association for the Advancement of Science.

Various mechanisms are employed to manipulate the fluorescence intensities or emission wavelengths of enzyme-responsive probes, including Förster resonance energy transfer (FRET), photo-induced electron transfer (PET), intramolecular charge transfer (ICT), etc. For example, FRET is a nonradiative process during which the energy of an excited state dye donor transfers to a ground state dye acceptor via dipole–dipole interactions [50]. Usually, an enzyme-responsive small molecular probe based on FRET is obtained by linking a fluorophore to its quencher with a specific peptide sequence, which could be cleaved by the enzyme to turn “On” the fluorescence of the fluorophore [51,52]. Furthermore, integrating a fluorescent dye and the corresponding quencher into a nanoprobe is also a promising method for achieving enzyme activity imaging. In 2020, Kulkarni and coworkers reported a granzyme B nanoreporter (**GNR**) comprising a FRET pair linked with a granzyme B cleavable peptide sequence [43]. **GNR** could deliver an immune checkpoint inhibitor (e.g., anti-programmed death-ligand 1 (PD-L1) antibody) to the highly immunogenic tumor, inducing the release of granzyme B to turn “On” the fluorescence of **GNR** for real-time imaging of immunotherapy response. However, in regard to poorly immunogenic tumors, the fluorescent signals remained in the “Off” state (Figure 1a), indicating that **GNR** was able to distinguish between highly responsive and poorly responsive tumors, as evidenced by the in vivo imaging results in Figure 1b. The authors also inoculated anti-PD-L1 sensitive (MC38) or insensitive (B16/F10) tumors into the right or left flank of a mouse, respectively. After intravenous (i.v.) injection of anti–PD-L1–coated **GNRs** (**αPDL1-GNRs**) or isotype control immunoglobulin G (IgG) antibody conjugated **GNRs** (**IgG-GNRs**), only anti-PD-L1 sensitive MC38 tumors treated with **αPDL1-GNRs** showed significantly enhanced NIR fluorescent signals.

**Figure 2:**
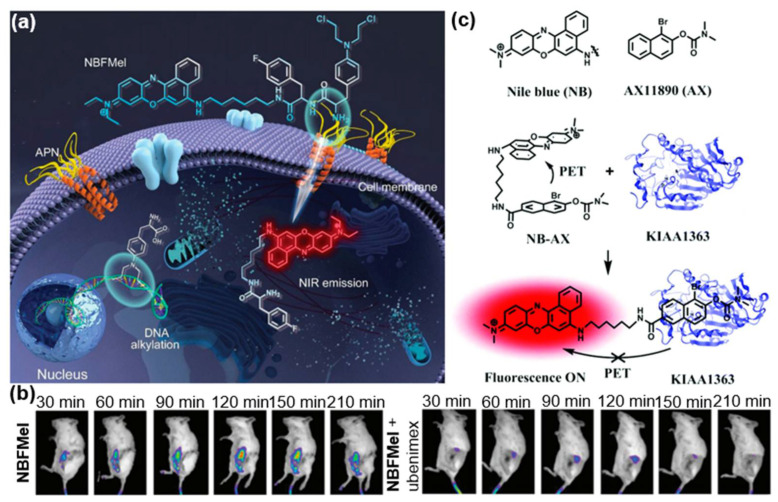
(**a**) Illustration of the mechanism of using prodrug **NBFMel** for tumor imaging and therapy. (**b**) Fluorescence imaging of B16/BL6 tumor-bearing mice treated with prodrug **NBFMel** or **NBFMel** with the APN inhibitor ubenimex. Reproduced with permission [54]. Copyright 2018, Wiley-VCH. (**c**) Chemical structure and responsive mechanism of **NB-AX**. Reproduced with permission [55]. Copyright 2017, Royal Society of Chemistry.

Other common enzyme-responsive fluorescence probes are based on PET, which typically contains an electron donor and acceptor connected by a short spacer leading to fluorescence quenching due to the intramolecular electron transfer [53]. Peng and coworkers reported an aminopeptidase N (APN)-activated nonfluorescent prodrug **NBFMel** [54]. Upon the cleavage of APN, free melphalan was released from the prodrug for tumor chemotherapy, and the PET process between melphalan and Nile blue within **NBFMel** was blocked to turn “On” the NIR fluorescence for tumor imaging (Figure 2a). A gradual increase in NIR fluorescence was observed in the B16/BL6 tumors in the mice i.v. injected with **NBFMel**, while the fluorescent signals were obviously suppressed in the tumor regions of the mice pretreated with the APN inhibitor ubenimex (Figure 2b). Moreover, the same group proposed a unique PET concept, folding PET, to design enzyme-activatable probes [55]. Adopting a flexible linear hexanediamine chain to link a NIR dye (Nile blue) and an inhibitor (AX11890) of the enzyme, a neutral cholesteryl ester hydrolase 1 (KIAA1363)-targeting probe **NB-AX** was developed for tumor imaging [45]. In the physiological environment, **NB-AX** adopted a folded conformation, which underwent a PET process between Nile blue and AX11890. When the AX11890 moiety was bound to KIAA1363, the probe would exhibit an unfolded conformation state to inhibit the PET process, thus switching “On” the NIR fluorescence of Nile blue (Figure 2c).

**Figure 3:**
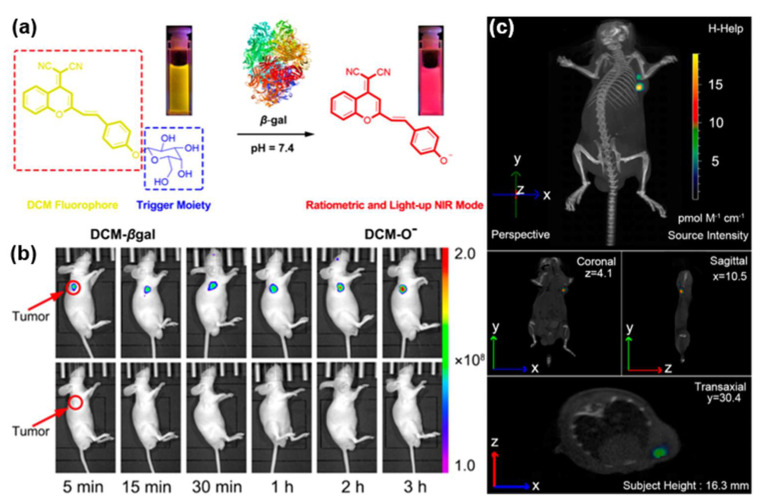
(**a**) Schematic illustration of β-galactosidase sensing mechanism of **DCM-*β*gal**. (**b**) Fluorescence imaging of LoVo tumor-bearing mice treated with Avidin-*β*-gal and **DCM-*β*gal** (top row) or only **DCM-*β* gal** (bottom row). (**c**) Three-dimensional in vivo imaging of the tumor-bearing nude mice treated with avidin-*β*-gal and **DCM-*β* gal**. Reproduced with permission [57]. Copyright 2016, American Chemical Society.

The ICT mechanism, which could modulate the emission wavelength of fluorophores, tends to be used for the design of ratiometric fluorescent probes [56]. In 2016, Zhu and coworkers developed an ICT NIR probe **DCM-*β*gal** by simply coupling dicyanomethylene-4*H*-pyran (DCM) chromophore with a β-galactosidase cleavable unit [57]. The oxygen atom in the β-galactosidase cleavage product of **DCM-*β*gal** (**DCM-O^-^**) served as a strong electron donor in the donor−π−acceptor (D−π−A) structure, leading to the increase in ICT and red shift of the maximum emission wavelength for ratiometric imaging of β-galactosidase in vivo (Figure 3a). The commercial tumor-targeting reagent avidin-*β*-gal i.v. injected into the tumor-bearing mice could efficiently target LoVo tumor cells and retain the enzymatic activity. After being pretreated with or without avidin-*β*-gal, the mice received the **DCM-*β*gal** injection. The tumor in the mice treated with avidin-*β*-gal and **DCM-*β*gal** exhibits brighter fluorescent signals than that in the mice only treated with **DCM-*β*gal** (Figure 3b,c). The in vivo imaging results further confirmed that **DCM-*β*gal** could be used for in vivo visualization of the activity of β-galactosidase.

**Figure 4:**
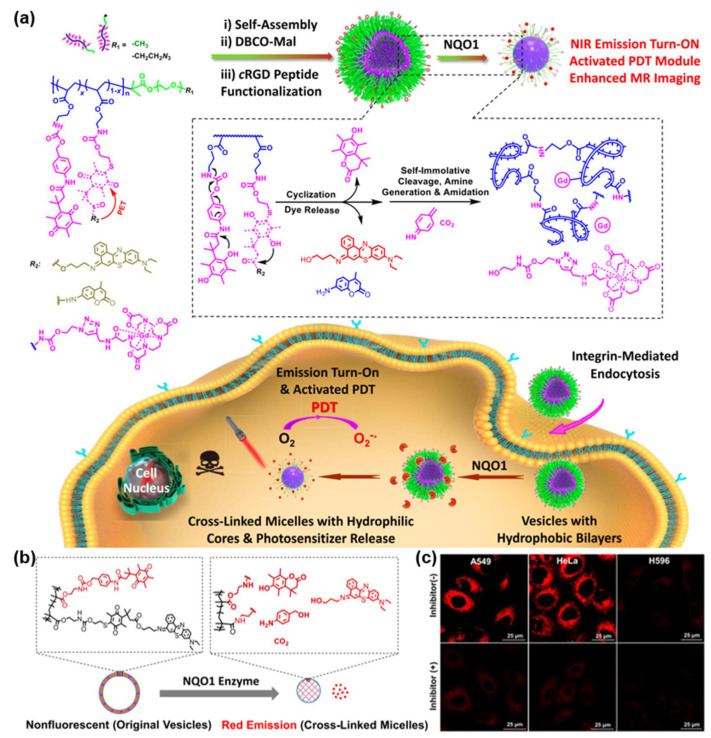
(**a**) Schematic illustration of NQO1-activated NIR fluorescence imaging and photodynamic therapy with polymeric vesicles. (**b**) The mechanisms of NQO1-triggered the release of NIR fluorophore Nile-blue. (**c**) Confocal laser scanning microscopy images of A549, HeLa, and H596 cells incubated with Nile-blue-labeled vesicles. The cells were pretreated with (bottom row) or without (top row) NQO1 inhibitor dicoumarol. Reproduced with permission [59]. Copyright 2020, American Chemical Society.

Fluorescent probes manipulated by multiple interplaying sensing mechanisms are also reported [58]. In 2020, Liu and coworkers reported the use of NAD(P)H:quinone oxidoreductase 1 (NQO1)-responsive photosensitizer-conjugated polymeric vesicles for tumor imaging and therapy [59]. The vesicles showed poor fluorescence and photodynamic property because of aggregation-caused quenching (ACQ) and photoinduced electron transfer (PET) effects. Upon NQO1-triggered self-immolative cleavage of the quinone linkages, free photosensitizers (e.g., Nile blue) were released from the vesicles to turn the NIR fluorescence “On” and activate photodynamic therapy (Figure 4a,b). During this process, the vesicles with hydrophobic bilayers were transformed into cross-linked micelles with hydrophilic cores, leading to the enhancement of magnetic resonance imaging when the vesicles were conjugated with a DOTA (Gd) complex (Figure 4a). Strong NIR fluorescence was observed from the NQO1-positive A549 and HeLa cells treated with NIR fluorophore Nile-blue-labeled vesicles without NQO1 inhibition in the cell imaging experiments (Figure 4c), suggesting Nile-blue could be released from the vesicles in NQO1-positive cells to turn “On” the fluorescence.

## 3. Enzyme-Instructed Self-Assembly of NIR Fluorophores In Vivo

**Figure 5:**
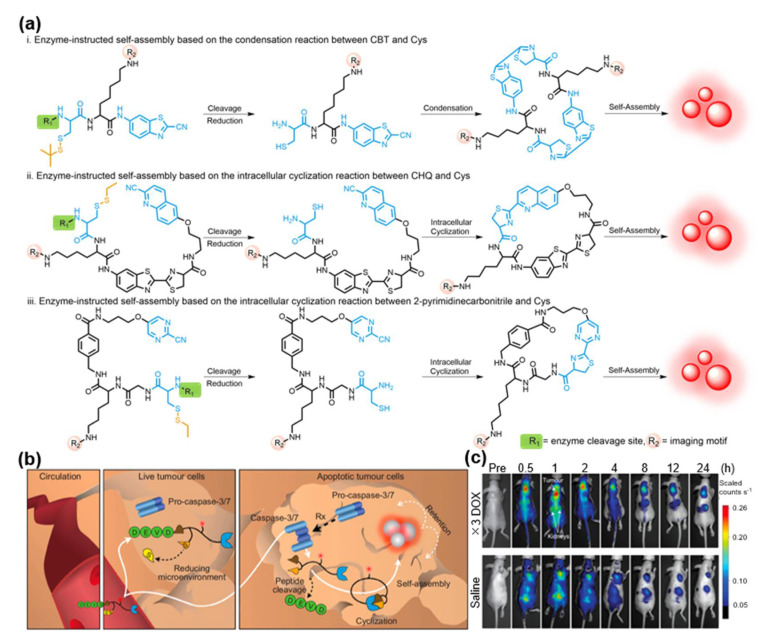
(**a**) Biocompatible condensation reactions between aromatic nitriles and amino thiols and their applications for enzyme-instructed self-assembly of imaging motifs for imaging. (**b**) Schematic illustration of **C**−**SNAF** for the imaging of apoptotic tumor cells. (**c**) Time−course fluorescence imaging of HeLa tumor-bearing mice. The mice were i.v. injected with **C**−**SNAF** three times for imaging after the treatment of DOX (top row) or saline (bottom row). The white arrows indicated tumors and kidneys. Reproduced with permission [63]. Copyright 2014, Nature Publishing Group.

Bioorthogonal click reactions [60], which occur rapidly without interference with biological processes in living systems, show great potential in the fabrication of probes characterized by enzyme-instructed self-assembly (EISA). Inspired by the synthesis of firefly _D_-luciferin, Rao and coworkers developed a biocompatible condensation reaction between 2-cyanobenzothiazole (CBT) and 1,2-aminothiol of cysteine (Cys) [61]. Under the control of pH, reduction, or enzyme, this reaction has been wildly explored for the synthesis of nanostructures in vivo for molecular imaging, cancer therapy, etc. (Figure 5a) [62]. However, due to its high reactivity, it is possible for CBT to react with abundant free cysteine in vivo. They further replaced CBT with 2-cyano-6-hydroxyquinoline (CHQ) to lower the reaction rate with free cysteine and developed a caspase-3/7-sensitive probe **C-SNAF** [63]. As shown in Figure 5b, after permeating the membrane of apoptotic tumor cell, **C-SNAF** underwent _L_-DEVD cleavage via activated caspase-3 and disulfide reduction to trigger intramolecular cyclization to form the macrocycle **C-SNAF-*cycl***, which further proceeded the in situ aggregation to yield nanoaggregates. The formation of these nanoaggregates allowed the NIR fluorophore Cy5.5 labeled on **C-SNAF** to accumulate inside cells, thus to image apoptotic tumors with high contrast. The fluorescence imaging of HeLa tumors indicated that **C-SNAF** showed brighter fluorescent signals in doxorubicin (DOX)-treated tumors than that in saline-treated, suggesting **C-SNAF** could be used for the monitoring of chemotherapy (i.e., DOX)-induced tumor apoptosis (Figure 5c). Later, they further altered the aromatic nitrile and aminothiol units, as well as the linking group between these two units, to further optimize the macrocyclization reaction and assembly process and finally chose the scaffold composed of 2-pyrimidinecarbonitrile and cysteine connected by a benzyl linker to design fluorescent probes for enzyme imaging in living cells (Figure 5a) [64].

**Figure 6:**
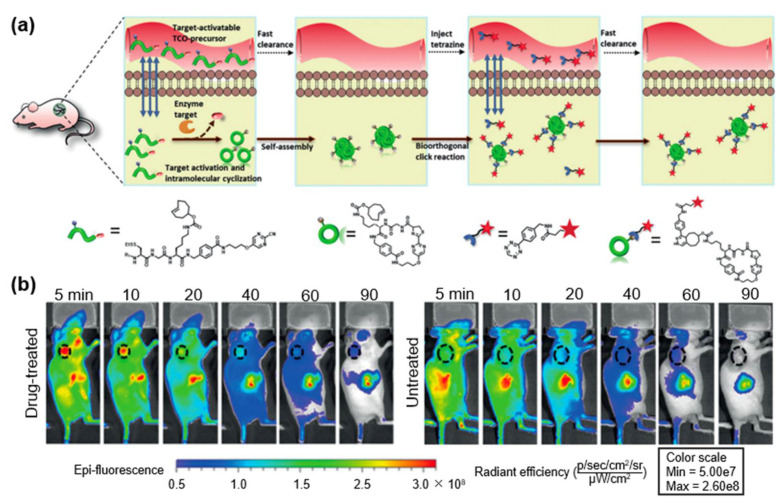
(**a**) Schematic illustration of the mechanism of pre-targeting strategy. The red star indicated the imaging unit such as Cy5 or DOTA-^64^Cu. (**b**) In vivo fluorescence imaging of H460 tumor-bearing mice consecutively injected with **TCO-C-SNAT4** and Tz-Cy5 after the treatment with cisplatin or not. The tumors were indicated with dotted oval. Reproduced with permission [65]. Copyright 2020, Wiley-VCH.

In the meanwhile, a pre-targeting strategy was developed for enzyme imaging using two bioorthogonal reactions [65]. Similarly, the handle probe **TCO-C-SNAT4** first underwent the condensation reaction of an aromatic nitrile and an aminothiol to self-assemble into *trans*-cyclooctene (TCO)-labeled nanoaggregates after being selectively activated by the cleavage of caspase-3/7 in apoptotic tumor cells. Then, the tetrazine (Tz)-modified imaging tag, such as Tz-Cy5 or Tz-DOTA-^64^Cu, was clicked onto the nanoaggregates retained at the target site via the inverse-electron demand Diels–Alder reaction (IEDDA) between Tz and TCO, generating selective fluorescence or positron emission tomography signals for enzyme imaging (Figure 6a). In the in vivo fluorescence imaging, the H460 tumor-bearing mice were pretreated with cisplatin to induce the activation of caspase-3/7 before the injection of **TCO-C-SNAT4**. After Tz-Cy5 injection, the tumor regions exhibited enhanced fluorescent signals compared with the mice without the treatment of cisplatin (Figure 6b), displaying the high efficiency of this pre-targeting strategy.

**Figure 7:**
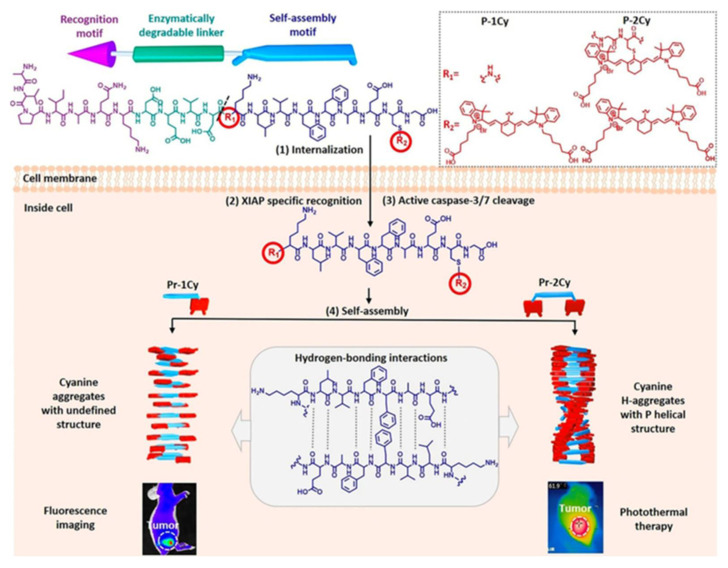
Schematic illustration of caspase-3/7-instructed self-assembly of peptide-cyanine conjugates with different cyanine dye arrangements for fluorescence imaging or photothermal therapy of tumors. Reproduced with permission [71]. Copyright 2021, Wiley-VCH.

Except for the above-mentioned self-assembly adopting covalent interactions and noncovalent interactions, other strategies are also developed to achieve in situ self-assembly only via noncovalent interactions. Peptides and their derivatives, which have been widely used in the development of diagnostic and therapeutic platforms, could form highly organized superstructures via noncovalent interactions (e.g., hydrogen bonding, π–π stacking, van der Waals interactions, and hydrophobic interactions) upon physical or chemical stimuli (e.g., enzymatic reactions) [66,67]. Previously, several NIR peptide probes using cyanine dyes as signaling molecules were developed, which targeted tumor cells and spontaneously self-assembled into nanofibers after the cleavage of enzymes, such as MMP-2/9 [68] and fibroblast activation protein-α (FAP-α) [69]. Generally, these peptide probes contained four parts: (1) a tumor-targeting motif, (2) an enzyme cleavage site, (3) a self-assembly motif, and (4) an imaging motif. These probes demonstrated a high SNR due to the aggregation/assembly-induced retention (AIR) effect [70], which could be used for image-guided surgery of renal cell carcinoma or small pancreatic tumor intraoperative imaging. To investigate the effect of the number of cyanine dyes on the molecular packing and the optical performances of nanofibers, Wang and coworkers designed two caspase-3/7-responsive peptide-cyanine conjugates with one or two cyanine substitutions, i.e., **P-1Cy** or **P-2Cy**, respectively [71]. The research showed that **P-1Cy** or **P-2Cy** could specifically recognize the X-linked inhibitor of apoptosis protein (XIAP), resulting in the activation of caspase-3/7. After being cleaved by the activated caspase-3/7, **P-1Cy** self-assembled into a loose column, and the cyanine dyes formed an undefined structure, which displayed excellent NIR fluorescence for tumor imaging. Nevertheless, **P-2Cy**, with two cyanine molecules, stacked into a P helical column with H-aggregated cyanine dyes under the same condition, leading to the enhancement of photothermal conversion efficiency for PTT of tumors (Figure 7).

**Figure 8:**
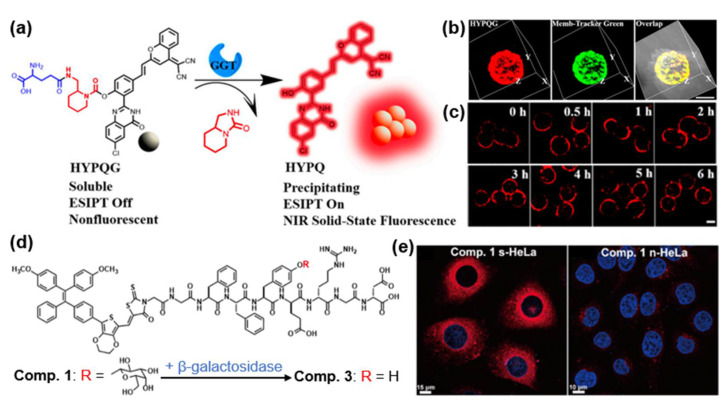
(**a**) The activation mechanism of **HYPQG** for GGT imaging. (**b**) Three-dimensional reconstructed images of A2780 cells incubated with **HYPQG**. The cell membrane was stained with Memb-Tracker Green. Scale bar, 20 μm. (**c**) Time-course fluorescence imaging of A2780 cells incubated with **HYPQG**. Scale bar, 20 μm. Reproduced with permission [72]. Copyright 2021, National Academy of Sciences. (**d**) Chemical structures of **Comp. 1** and **Comp. 3**. (**e**) Merged fluorescence images of senescent HeLa (s-HeLa) cells and normal HeLa (n-HeLa) cells incubated with **Comp. 1**.DAPI (blue), nuclear counterstaining. Red fluorescence, NIR fluorophore in **Comp. 1**. Reproduced with permission [73]. Copyright 2020, Science China Press and Springer-Verlag GmbH Germany, part of Springer Nature.

Moreover, some fluorophores can generate strong fluorescence in aggregation or solid states, showing great potential in the construction of self-assembly fluorescence probes. For instance, Zhang and coworkers reported a γ-glutamyltranspeptidase (GGT)-activated probe **HYPQG** based on a NIR precipitating solid-state fluorochrome (Figure 8a) [72]. Due to the excited-state intramolecular proton transfer (ESIPT) mechanism, **HYPQG** was nonfluorescent. The research showed that **HYPQG** was subjected to γ-glutamyltranspeptidase (GGT)-initiated release of free fluorophore **HYPQ**, which further precipitated at the tumor cell surface with a strong NIR fluorescence emission for long-term bioimaging of GGT on the living cell membrane. Cell experiments showed that when **HYPQG** was incubated with GGT-overexpressing A2780 tumor cells, the red fluorescence of **HYPQ**, GGT cleavage product of **HYPQG**, co-localized well with the green fluorescence of commercially available cell membrane tracker (i.e., Memb-Tracker Green) (Figure 8b) and remained unchanged for 6 h (Figure 8c), indicating the antidiffusion property of **HYPQG** for long-term in situ imaging of GGT. Meanwhile, from the employment of EISA strategy and NIR fluorophores with aggregation-induced emission (AIE) property, a novel NIR fluorescent probe **Comp. 1** was developed, which could interact with senescence-associated β-galactosidase to yield **Comp. 3** and self-assemble into nanofibers with enhanced NIR fluorescence (Figure 8d) [73]. Cell imaging experiments, as shown in Figure 8e, demonstrated that **Comp. 1** displayed a brighter fluorescence when incubating with senescent tumor cells than with normal tumor cells. Moreover, the as-formed nanofibers exhibited high photodynamic therapy efficiency, suggesting that **Comp. 1** could be used for the selective detection and removal of senescent tumor cells.

## 4. Enzyme-Instructed Disassembly of NIR Fluorophores In Vivo

**Figure 9:**
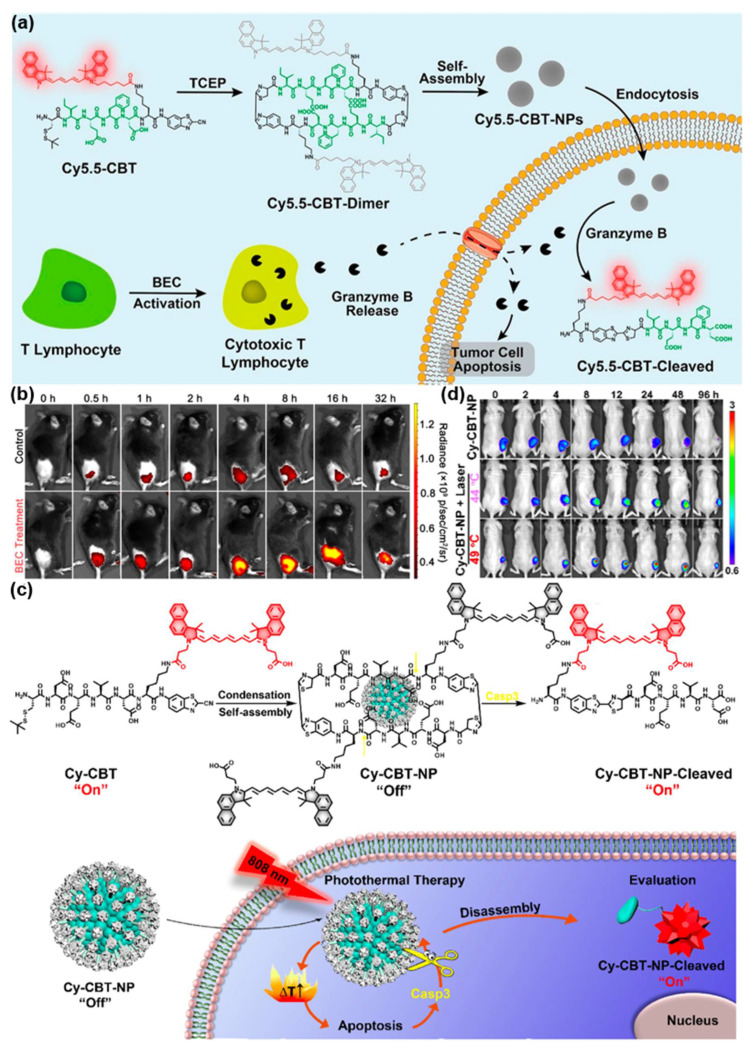
(**a**) Illustration of the fabrication of **Cy5.5-CBT-NPs** and its application for the evaluation of the cancer immunotherapy efficacy. (**b**) Time-course fluorescence imaging of B16-OVA tumor-bearing mice pretreated with saline or an immunotherapeutic agent *S*-(2-boronoethyl)-*L*-cysteine hydrochloride (BEC) before the intratumoral injection of **Cy5.5-CBT-NPs**. Reproduced with permission [44]. Copyright 2022, American Chemical Society. (**c**) Schematic illustration of **Cy-CBT-NP** for real-time self-evaluation of the PTT efficiency. (**d**) Fluorescence imaging of **Cy-CBT-NP**-treated mice after irradiation with 808 nm laser for different times to reach different temperatures at the tumor regions (44 °C for mild apoptosis, middle row; 49 °C for severe apoptosis, bottom row). The mice without laser irradiation (top row) were used as control. Reproduced with permission [77]. Copyright 2020, American Chemical Society.

Liang and coworkers developed an enzyme-instructed disassembly strategy for NIR fluorescence “Turn-On” imaging of tumors. Specifically, a NIR dye (e.g., Cy5.5) labeled CBT derivative was synthesized and assembled into nanoparticles via reduction (e.g., tris(2-carboxyethyl) phosphine (TCEP))-controlled CBT-Cys click reaction, which led to the fluorescence quenching due to ACQ effect. After being translocated into tumor cells, the nanoparticle was disassembled by the cleavage of intracellular abundant enzymes, such as furin [74], protease [75], or legumain [76], resulting in the recovery of the NIR fluorescence for tumor imaging. Subsequently, they expanded the applications of this in vivo disassembly strategy depending on the enzymes correlated with physiological and pathological processes. For example, to evaluate the efficacy of cancer immunotherapy, they reported Cy5.5 fluorescence-quenched nanoparticles **Cy5.5-CBT-NPs** for NIR fluorescence “Turn-On” imaging of granzyme B [44], a biomarker associated with immunoactivation (Figure 9a,b). Additionally, they developed nanophotothermal transduction agents (PTAs) **Cy-CBT-NP** with absorption in NIR windows for PTT and self-evaluation of the therapeutic efficiency of tumors [77]. As shown in Figure 9c, after entering tumor cells, **Cy-CBT-NP** displayed an excellent photothermal effect under 808 nm laser irradiation, which activated caspase-3 and eventually led to tumor cell death. In turn, the activated caspase-3 could disassemble **Cy-CBT-NP** to turn “On” the NIR fluorescence, which could be used for the real-time evaluation of the PTT efficiency. The brightness of the NIR fluorescence from the tumor region was positively correlated with the PTT efficiency, i.e., the temperature of the tumor received different treatments (Figure 9d). Except for in vitro assembled nanoparticles based on CBT-Cys click reaction, other nanoprobes, such as polymer or borondipyrromethene-based nanoparticles [78,79], were also developed for enzyme imaging.

**Figure 10:**
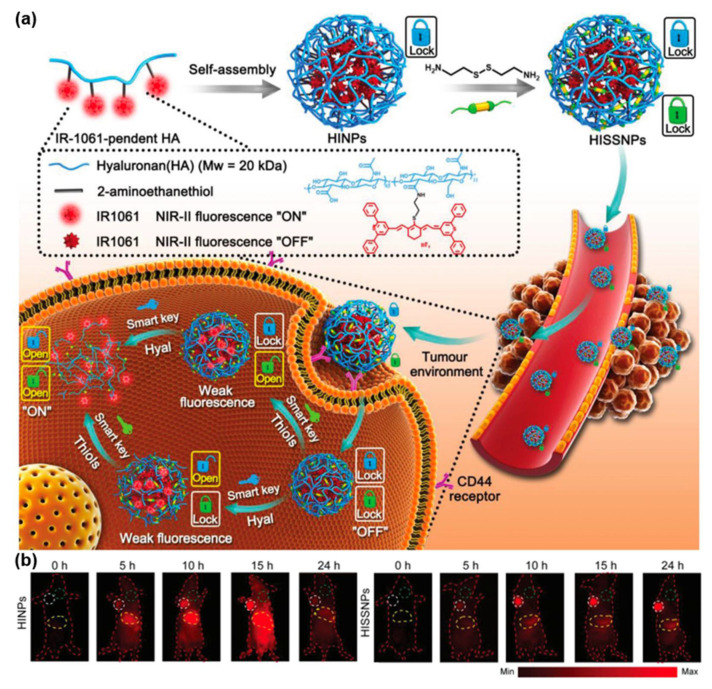
(**a**) Schematic illustration of “dual lock-and-key” strategy for tumor-specific imaging. (**b**) In vivo fluorescence imaging of MCF-7 tumor-bearing mice injected with **HISSNPs** or **HINPs**. Reproduced with permission [80]. Copyright 2018, Wiley-VCH.

Nevertheless, the above-mentioned nanoparticles aimed at a single target (i.e., a specific enzyme), might cause nonspecific activation and even “false positive” result due to the complex and dynamic physiological and pathological environment. To deal with this problem, Fan and coworkers reported hyaluronidase and thiols cooperatively activatable nanoprobes **HISSNPs** for ultrahigh-specific imaging of tumors in vivo [80]. Briefly, hydrophobic NIR dyes IR-1061, hydrophilic hyaluronic acid (HA), and disulfide were used to construct “dual lock-and-key”-controlled nanoparticles **HISSNPs**. The quenched fluorescence of **HISSNPs** would be turned “On” only when hyaluronidase and thiols were excited simultaneously (Figure 10a). Compared with “single lock-and-key”-controlled nanoparticles (**HINPs**) without the modification of disulfide, **HISSNPs** displayed lower background, higher tumor-to-background ratio, and tumor-to-liver ratio, realizing tumor imaging with ultrahigh specificity (Figure 10b).

## 5. Conclusions

In summary, we have enumerated the representative examples of enzyme-activatable NIR fluorescent probes, including the following respects: enzyme-instructed release of free NIR fluorophores in vivo and enzyme-instructed self-assembly/disassembly of NIR fluorophores in vivo. In particular, the design mentalities and the sensing mechanisms are also discussed. Generally, upon interaction with an enzyme, a free fluorophore could be released from the probe to change the FRET, PET, or ICT process for “Turn-On” or “Ratiometric” fluorescence imaging. Enzyme-instructed self-assembly allowed higher accumulation and prolonged retention time of NIR fluorophores in situ, which successfully improved NIR fluorescent signals at the target sites. Meanwhile, the ACQ effect was usually employed to construct a fluorescence-quenched nanoprobe. The cleavage of an enzyme could destroy the nanostructure and trigger the fluorescence from the “Off” to “On” state.

Despite the significant progress that has been made in this field, enzyme-activatable NIR fluorescent probes still face large challenges. (1) The vast majority of reported enzyme-activatable NIR fluorescent probes had fluorescence emission in the NIR-I window. As NIR-II fluorescence shows a higher tissue penetration and SNR than that of NIR-I [81], the development of enzyme-activatable probes with fluorescence emission in the NIR-II region is attractive and has wide application prospects in disease diagnosis [82,83]. However, there are few publications about enzyme-activatable probes using NIR-II fluorophores as imaging motifs. (2) The up- or down-regulation of a certain enzyme is closely related to the disease progression. For example, alkaline phosphatase exists in all tissues of the entire body, which shows higher levels in the blood of patients with critical diseases such as liver damage, bile cancers, or heart failure and lower levels in the blood of people suffering from malnutrition [84]. Non-invasive dynamic evaluation of the activity of the enzyme is of great significance to determine the progression and the therapeutic efficiency of the disease. But there still exist some difficulties in the in vivo real-time quantification of the expression level of an enzyme via fluorescence imaging. For instance, the fabrication of fluorescence probes with fast and reversible responses toward enzymes to image the dynamic changes remains tough. Moreover, some enzymes are present in many normal tissues. How to avoid interference from normal tissues and identify the subtle changes, especially the down-regulation of the enzymes at target sites, is considered one of the key problems for in vivo real-time quantification. (3) The accumulation and elimination of a probe also need to be considered. The high accumulation of probes at target sites is essential to obtain images with high quality and resolution through i.v. injection. In situ self-assembly strategy would improve the accumulation, but it is still not enough. Most fluorescent probes i.v. injected into the body were captured via the reticuloendothelial system (RES), resulting in a low accumulation of probes at lesions and high imaging background [85]. The combination of a self-assembly strategy with efficient and precise targeting motifs might improve the accumulation efficiency of the probes. At the same time, the elimination of a probe is associated with biosafety. The in vivo disassembly of a probe with a large size might be profitable for the elimination. Probes integrated with clearance moieties could also lead to fast elimination. More attention should be paid to the time window of accumulation and elimination to achieve imaging with high SNR and biosafety.

There are still significant opportunities and challenges in developing ideal fluorescent probes, and great efforts should be made to promote clinical translation. With their unique merits, we believe that enzyme-activatable NIR fluorescent probes will provide new possibilities for unprecedented and exciting applications in various fields.

## Data Availability

Not applicable.
